# *In vivo* studies of the *Drosophila* insulator factor CTCF reach a Catch 22

**DOI:** 10.1186/s12915-015-0182-9

**Published:** 2015-09-02

**Authors:** François Karch

**Affiliations:** Department of Genetics and Evolution, University of Geneva, 30 quai Ernest Ansermet, 1211 Geneva, Switzerland

## Abstract

Mutations in the proteins that bind insulator DNA elements that define the boundaries of chromatin domains can give morphogenetic readouts in *Drosophila*, as recently reported in BMC Biology by Bonchuk *et al.* in the Georgiev laboratory. But disentangling the effects on the phenotype may not be simple.

See research article: http://www.biomedcentral.com/1741-7007/13/63

## Insulator/boundary elements

Insulator DNA elements, also referred to with the more generic term boundaries, are important architectural components of the genome and nuclear organization that are conserved through evolution. They play an active part in gene regulation by binding proteins that interact to organize chromatin into loops thought to define autonomous chromatin domains (reviewed in [1, 2]), thereby modulating the activities of enhancers and silencers in specific tissues and/or developmental stages. They were first identified in *Drosophila* on the basis of their enhancer-promoter blocking activity, an activity thought to restrict the promiscuous activity of enhancers. Insulators also protect transgenes from genomic position effects, by establishing independent functional domains within the chromosomes (for review see [1]). Bonchunk *et al*. [3] now report a combination of biochemical and genetic experiments with CTCF, the most conserved DNA-binding insulator protein, in *Drosophila*. Thanks to the powerful *Drosophila* molecular genetic toolbox, the new biochemical properties of CTCF described in the paper are directly assessed *in vivo*. I believe that these new *in vivo* results reach a Catch 22 situation that complicates the interpretation and that is the subject of this commentary.

## Proteins associated with insulator boundaries

The proteins that associate with boundaries/insulators fall into two categories. The first group encompasses the DNA-binding proteins. Here most of what we know comes from studies in *Drosophila* where nine of them have been identified. They include in the order of their identification Su(Hw), BEAF-32, the GAGA factor, Zw5, dCTCF, the Elba1/Elba2/Elba3 protein complex, Pita and ZIPIC, and finally the Ibf1 and Ibf2 proteins (see original citations in [3]). Unexpectedly, the situation is apparently much simpler in vertebrates where only CTCF has been identified as an insulator DNA binding protein (for review see for example [2]; the other fly proteins do not have obvious orthologs in vertebrates). ChiP on ChiP/seq analysis revealed the existence of multiple genomic sites with distinct combinations of these insulator DNA-binding factors (see for example [4] and references in [3]).

The second group of proteins that associate with *Drosophila* insulators consists of CP190, and multiple protein isoforms encoded by the locus mod(mdg4) (see [1, 5, 6] for references to original papers). Cp190 and Mod(mdg4) do not bind directly to DNA but they can mediate homotypic and heterotypic protein-protein interactions via their BTB/POZ domains. Interestingly these two proteins are associated with most boundary/insulator elements within the genome, and CP190 was proposed to play the role of universal ‘glue’ that mediates long-distance interactions between insulator elements of different classes, thereby generating chromatin loops [4]. In vertebrates, the glue component associating with CTCF is thought to be the cohesin complex [7].

## dCTCF protein and biology

Vertebrate and *Drosophila* CTCF are well conserved in their 12 C2H2 zinc finger domains, and bind similar sequences [8]. In contrast their N- and C-terminal domains differ, probably reflecting their different interaction partners (see above). Using various biochemical methods, Bonchuk *et al*. [3] find a multimerization domain in the N-terminus of dCTCF that likely mediates the formation of tetrameric complexes. At the C-terminal domain a module encompassing 200 amino acids of dCTCF mediates interaction with the N-terminal BTB domain of CP190.

In trying to study the functional significance of these two domains *in vivo*, the authors made interesting observations. First they reassessed the phenotype of various CTCF alleles available in the literature [6] and provide compelling evidence that a null mutation is able to develop to adulthood. This does not necessarily imply that dCTCF is dispensable for development however. Many essential genes are heavily transcribed during oogenesis and their transcripts are transferred to the oocyte, allowing development to proceed until adulthood thanks to the maternal store. The more surprising observation is that normal oogenesis can proceed in homozygous null dCTCF females, which lay eggs able to undergo normal development if they are fertilized by a sperm carrying a functional dCTCF gene.

Among various hypotheses discussed in the article I like the idea that fly insulators are the targets of multiple DNA binding proteins, allowing some functional redundancy: in this view the lack of one insulator protein can be compensated for by an alternative one. Redundancy may also explain why a CTCF protein lacking the CP190 interaction domain is almost as effective as the wild-type form of CTCF in rescuing the dCTCF mutant phenotype. Indeed CP190 can be recruited by an alternative insulator protein such as Su(Hw), Ibf1/Ibf2,, Pita or ZIPIC (see ref 3 for references to the original papers). In contrast, a CTCF protein lacking the multimerization domain is less efficient in rescuing the dCTCF mutant phenotype, opening up the possibility that CTCF multimer formation is important for insulator binding.

## Boundary mutations in the bithorax-complex (BX-C)

While homozygous null dCTCF flies can develop to adulthood they do not look entirely wild type, harboring thin bristles, partially folded wings and homeotic transformations of the posterior abdominal segments (see below). The question then arises of whether the phenotypes observed can shed some light on insulator or boundary functions. The question is not trivial to address because nearly all insulators/boundaries have been identified with reporter gene assays, and thus outside their native chromosomal context. An answer to this problem comes from a special class of gain-of-function (GOF) mutations in the bithorax complex of hox genes (BX-C) that cause homeotic transformations of abdominal segments. These dominant mutations result from the deletions of boundaries. Very interestingly, dCTCF homozygotes show homeotic transformations of the posterior abdominal segments that are specified by the *Abd-B* gene of the BX-C, suggesting that the boundary functions in the BX-C are compromised in dCTCF mutant background.

**Fig. 1.**
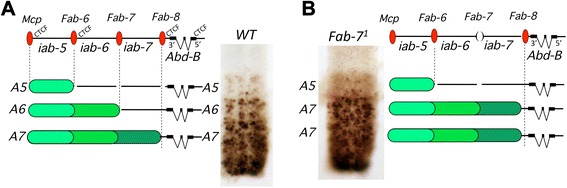
The *Abd-B* gene and its associated 3’ regulatory region (not drawn to scale) cover 165 kilobases of DNA. See text for explanation. Wild type (WT) (**a**) and *Fab-7* ^*1*^ (**b**) embryonic nerve cords stained with antibodies against *Abd-B* have been dissected out. Abdominal segments are numbered A5, A6, A7. Panel (**a**) shows the presence of CTCF binding sites at *Mcp, Fab-6, Fab-8* and at the *Abd-B* promoter. The 5’-3’ polarity of the *Abd-B* transcription unit is indicated

Fig. 1a depicts the regulatory landscape of the *Abd-B* gene that specifies the identity of the fifth to the eighth abdominal segments (A5 to A8). *Abd-B* is expressed in a graded fashion from A5 to A8 (as shown in the embryonic nerve chord, Fig. 1). This graded expression from anterior to posterior (also the case for the two other hox genes of the BX-C, *Ubx* and *abd-*A) is at the basis of a general rule for the behavior of homeotic mutations in the BX-C: gain-of-function (GOF) mutations result in the transformation of a given segment towards the identity of the more posterior segment, while loss-of-function (LOF) mutations lead to the opposite homeotic transformation, towards a more anterior segment (for review see [9]). The *Abd-B* segment-specific expression in A5, A6 A7 and A8 is controlled by the large regulatory domains *iab-5*, *iab-6*, *iab-7* and *iab-8* respectively (Fig. 1a; the *iab-8* regulatory domain is not shown for simplicity). Activation of the segment-specific regulatory domains is thought to occur through sequential opening of chromosome domains as drawn in Fig. 1a and b (for review see [9]). The extent of each regulatory domain is defined by boundaries (*Mcp*, *Fab-6. Fab-7* and *Fab-*8). As an example, Fig. 1b depicts the consequence of a deletion of the *Fab-7* boundary that separates *iab-6* from *iab-7*. Upon opening of *iab-6* in A6, the active state spreads towards *iab-7*, leading to its activation one segment too anterior. As a result, *Abd-B* is expressed in the A7-like expression pattern in A6 resulting in the GOF homeotic transformation of A6 into A7. Occasionally, the inactive state of *iab-7* in A6 can prevail and invade *iab-*6. The A6 cells in which this occurs acquire an A5 identity (LOF phenotype; see [3] for references to the original work).

Additional boundaries flank the regulatory domains that control *abd-A* in the more anterior abdominal segments A2-A4. Interestingly, all BX-C boundaries (with the exception of *Fab-*7) contain binding sites for dCTCF [8]. In a simple world, mutation in dCTCF would be expected to interfere with all BX-C boundaries and result in an all out situation in which all regulatory domains open up simultaneously. In the case of *Abd-B,* an A8-like expression pattern should appear from A5, resulting in a posterior-oriented transformation of segment identity (towards A8). The reality is more complex, with dCTCF null homozygous flies harboring a mixture of GOF and LOF phenotypes in the identities of their posterior abdominal segments. Bonchunk *et al*. favor the idea that this mixture of GOF and LOF phenotypes is a readout of crippled boundaries that would either fail to block activation spreading (GOF) or silencing spreading (LOF).

But this mixture of GOF and LOF transformation of segment identity may equally reflect a difficulty of interpretation inherent in the multifarious nature of CTCF, which can bind both to the insulators and to the *Abd-B* promoter. Thus in a dCTCF mutant background, a loss of activity of the *Abd-B* promoter may also occur in addition to the crippled boundaries. In as much as *Abd-B* activity is required for the readout of boundary mutations, one reaches a sort of Catch-22 situation. Some cells may have enough CTCF to ensure proper *Abd-B* promoter activity to display the GOF phenotype, while some other cells may shut down their *Abd-B* promoter, leading to a LOF phenotype. A similar scenario could explain conflicting observations made with the GAGA factor, which according to different authors is classified either as trithorax-group genes (trx-G, activators [10]) or as Polycomb-group genes (Pc-G, repressors [11]. On one hand, the Pc-G activity of the GAGA factor was concluded from experiments performed ectopically using a reporter gene assay. On the other hand, the trx-G activity of GAGA was based on the LOF homeotic phenotype observed in the context of a GAGA mutant background. As for CTCF, GAGA sites also decorate the Hox gene promoters. A compromised Polycomb repression remains ineffective if the promoter for its readout is also inactive.

## A safety net for chromatin organizing factors

Somehow multi-tasking proteins, such as CTCF or GAGA, that bind at both regulatory elements and at the target promoter controlled by these regulatory elements, are difficult to study by genetics. Genes controlling development are often expressed in very tight spatio-temporal patterns that are controlled by complex and extensive cis regulatory regions. Any defects in the organization of these control regions may have important effects on the expression pattern and lead to deleterious effects. Having the factors organizing the activity of these regulatory regions also involved in promoter activity may have been adapted though evolution to buffer the consequences of mis-regulation.

## References

[1] Chetverina D, Aoki T, Erokhin M, Georgiev P, Schedl P (2014). Making connections: insulators organize eukaryotic chromosomes into independent cis-regulatory networks. Bio Essays.

[2] Ong CT, Corces VG (2014). CTCF: an architectural protein bridging genome topology and function. Nat Rev Genet.

[3] Bonchuk A, Maksimenko O, Kyrchanova O, Ivlieva T, Mogila V, Deshpande G (2015). Functional role of dimerization and CP190 interacting domains of CTCF protein in Drosophila melanogaster. BMC Biol.

[4] Schwartz YB, Linder-Basso D, Kharchenko PV, Tolstorukov MY, Kim M, Li HB (2012). Nature and function of insulator protein binding sites in the Drosophila genome. Genome Res.

[5] Gerasimova TI, Gdula DA, Gerasimov DV, Simonova O, Corces VG (1995). A Drosophila protein that imparts directionality on a chromatin insulator is an enhancer of position-effect variegation. Cell.

[6] Mohan M, Bartkuhn M, Herold M, Philippen A, Heinl N, Bardenhagen I (2007). The Drosophila insulator proteins CTCF and CP190 link enhancer blocking to body patterning. EMBO J.

[7] Wendt KS, Yoshida K, Itoh T, Bando M, Koch B, Schirghuber E (2008). Cohesin mediates transcriptional insulation by CCCTC-binding factor. Nature.

[8] Holohan EE, Kwong C, Adryan B, Bartkuhn M, Herold M, Renkawitz R (2007). CTCF genomic binding sites in Drosophila and the organisation of the bithorax complex. PLoS Genet.

[9] Maeda RK, Karch F (2006). The ABC of the BX-C: the bithorax complex explained. Development.

[10] Farkas G, Gausz J, Galloni M, Reuter G, Gyurkovics H, Karch F (1994). The Trithorax-like gene encodes the Drosophila GAGA factor. Nature.

[11] Hagstrom K, Muller M, Schedl P (1997). A *Polycomb* and GAGA dependent silencer adjoins the *Fab-7* boundary in the *Drosophila* bithorax comple. Genetics.

